# Vestibular and proprioceptive contributions to trunk stabilization differ across postural tasks and walking speeds

**DOI:** 10.1242/jeb.251082

**Published:** 2026-04-21

**Authors:** Yiyuan C. Li, Sjoerd M. Bruijn, Koen K. Lemaire, Simon Brumagne, Jaap H. van Dieën

**Affiliations:** ^1^ Department of Human Movement Sciences, Faculty of Behavioural and Movement Sciences, Vrije Universiteit Amsterdam, Amsterdam Movement Sciences, 1081 BT Amsterdam, The Netherlands; ^2^ Department of Rehabilitation Sciences, KU Leuven, 3001 Leuven, Belgium

**Keywords:** Postural control, Gait, Standing, Vestibular afference, Proprioception

## Abstract

Stabilizing upright posture of the trunk in humans relies on vestibular and proprioceptive afference. Previous studies found that the feedback responses to sensory afference vary between postures and tasks. We investigated whether and how vestibular and proprioceptive afference contribute to trunk stabilization during different postural tasks, and during walking at different speeds. Twelve healthy adults performed tasks in a random order – sitting, standing on the right foot or both feet, and treadmill walking at five speeds (0.8, 2.0, 3.2, 4.3 and 5.5 km h^−1^) – while exposed to unilateral muscle vibration on the right paraspinal muscles at the level of the second lumbar vertebra, or to a step-like electrical vestibular stimulation (EVS) with the anode behind the left ear. The mediolateral displacements of markers at the sixth thoracic (T6) level and the sacrum in the global coordinate system were used to evaluate the responses to sensory stimulation. No significant responses to EVS at the T6 and sacrum levels were found in sitting and standing. Responses to muscle vibration were significant and differed between unipedal standing compared with sitting and bipedal standing. The latter suggests a different interpretation of the sensation of muscle lengthening in these postures. During walking, the magnitude of the responses to both stimuli increased from very slow speeds to moderate speeds. From moderate to higher speeds, responses to muscle vibration decreased, whereas responses to EVS plateaued. These findings suggest speed-dependent modulation of vestibular and proprioceptive contributions in trunk stabilization during walking.

## Teaser

**Summary:** Application of electrical vestibular stimulation and muscle vibration shows that vestibular and proprioceptive signals differ in how they are used for trunk stabilization between postural tasks and walking speeds.

## INTRODUCTION

The trunk represents approximately 50% of the total body mass in humans (de Leva, 1996). With its location high above the base of support, a small deviation from the upright position can produce a substantial destabilizing moment. Trunk muscles have an active role in maintaining the upright posture of the trunk and head (Hof, 2007; Li et al., 2024; Wu et al., 2019), which supports the use of visual and vestibular information for postural control.

Feedback information for the stabilization of upright posture against gravity is provided by different sensory modalities (Asslander and Peterka, 2014; Maurer et al., 2006; Peterka, 2002; Peterka and Loughlin, 2004). For example, vestibular signals contribute to perception of the head orientation in space (Cullen, 2012), signals from muscle spindles provide a sense of trunk orientation and movement (Prochazka, 2021), and visual information is used to assess the direction and speed of self-motion (Gibson, 1958). These signals need to be integrated to obtain estimates relevant for stabilization of upright posture but are also to some extent redundant. It is believed that redundant sensory signals are integrated based on their relative reliability, in a process called ‘sensory reweighting’ (Asslander and Peterka, 2014; Goar et al., 2025; Oie et al., 2002; Peterka and Loughlin, 2004). This reweighting process compensates for imprecision, loss or interruption of input from one or more sensory modalities.

The contribution of each sensory modality to stability control is commonly investigated with sensory perturbations (Alberts et al., 2019; Anson et al., 2014; Courtine et al., 2007; Day, 1999; Duclos et al., 2014; Li et al., 2024). For instance, the contribution of vestibular information has been investigated with electrical vestibular stimulation (EVS) (Alberts et al., 2019; Day, 1999; Li et al., 2024). The contribution of proprioceptive signals has been investigated with muscle vibration, causing an illusion of muscle lengthening (Goodwin et al., 1972; Inglis et al., 1991; Vizirgianakis et al., 2021).

Previous studies suggest that the gain of feedback responses to sensory afference may depend on the task performed or posture adopted. It is likely that this task- or posture-specific effect of sensory feedback relates to the difficulty of stabilizing these postures (Ali et al., 2003; Britton et al., 1993; Day et al., 1997; Fitzpatrick et al., 1994; Grangeon et al., 2015; Vizirgianakis et al., 2021). For example, in sitting (with a larger base of support), responses to visual, vestibular and proprioceptive perturbations are significantly smaller than in standing (Ali et al., 2003; Grangeon et al., 2015; Vizirgianakis et al., 2021). Additionally, EVS-induced responses were found to decrease with increased stance width and with the presence of external support (Britton et al., 1993; Day et al., 1997; Fitzpatrick et al., 1994). In bipedal standing, responses to visual perturbations were significantly smaller compared with unipedal standing, a more challenging posture (Hazime et al., 2012). However, Hazime et al. (2012) reported that ankle vibration had less effect in unipedal standing than in bipedal standing. They assumed that the vestibular signal compensates for the decreased use of proprioception through sensory integration (Hazime et al., 2012). This suggests that posture- or task-dependent effects may, in part, reflect reweighting instead of an overall change in feedback gain. From this perspective, the decreased effect of ankle vibration could also be attributed to decreased proprioceptive input from the ankle being compensated by an increased input from the hip and trunk, because corrective actions from the hip and trunk are required to maintain upright posture during unipedal standing (Riemann et al., 2003).

Unlike in standing, which needs continuous stabilization, the contribution of sensory signals to stabilization of walking is phasic, as reflected by phase-dependent gains of feedback responses (Blouin et al., 2011; Ivanenko et al., 2000; Li et al., 2024; O'Connor and Kuo, 2009). The use of sensory signals in stabilization of walking is affected by walking speed; smaller effects of vestibular stimuli were found at higher compared with lower walking speeds (Brandt et al., 1999; Dakin et al., 2013; Fabre-Adinolfi et al., 2018; Jahn et al., 2000; Schniepp et al., 2012). Visual perturbation by means of optic flow was found to have smaller effects in running than in walking, also suggesting a lower contribution of visual signals at higher speeds (Jahn et al., 2001). Based on sensory reweighting, signals from one or more alternative modalities may compensate for the decreased contributions of vestibular and visual signals to stability control during faster walking. Proprioceptive signals could be a potential source of compensation in fast walking, as it has been shown that after removal of proprioceptive feedback, mice maintained the ability to walk at slower speeds but failed to walk at higher speeds (Mayer et al., 2018; Takeoka et al., 2014). However, to the best of our knowledge, similar findings have not yet been reported in humans. Additionally, these studies only included limited speed ranges, i.e. comparing walking at a self-selected speed with running, or walking at a higher speed.

Here, we studied whether and how proprioceptive and vestibular signals contribute to trunk stabilization during sitting, unipedal standing, bipedal standing and during walking at different speeds, using muscle vibration on lumbar paraspinal muscles and EVS. We extended the range of walking speeds tested in previous studies to very slow speeds. We focused on the initial responses to step-like sensory stimulation, as later responses will be more affected by the gravitational perturbation that may follow from compensatory movements, and this effect varies between postures. We hypothesized that compared with bipedal standing, during unipedal standing, which poses a greater challenge to stability, both EVS and muscle vibration would lead to larger responses. Similarly, during sitting, which is inherently more robust with a larger base of support, the responses to EVS and muscle vibration would be smaller than during bipedal standing. During walking, EVS was expected to elicit larger responses at slower speeds as reported previously, whereas muscle vibration was expected to elicit larger responses at faster walking speeds because of sensory reweighting.

## MATERIALS AND METHODS

### Participants

Nineteen healthy adult humans were recruited. Seven participants did not complete all measurements owing to a damaged muscle vibrator. In total, data from 12 participants were used in this study (*N*=12, 7 males, 5 females; age: 19.9±2.1 years; mass: 65.7±7.5 kg; height: 1.72±0.09 m). Exclusion criteria for participation were any diagnosed orthopaedic or neurological disorders, or the use of medications that can cause dizziness. All participants provided written informed consent. Procedures were approved by the VU Amsterdam Research Ethics Committee (VCWE-2023-136).

### Electrical vestibular stimulation

EVS was applied as an analogue signal through a digital-to-analogue converter (National Instruments Corp., Austin, TX, USA) to an isolated constant-current stimulator (BIOPAC System Inc., Goleta, CA, USA). The current was delivered via two carbon rubber electrodes (9 cm^2^), which were coated with electrode gel (SonoGel, Bad Camberg, Germany) and placed over the mastoid processes with the anode on the left and cathode on the right. In this configuration, a step-like EVS signal, with an amplitude of 1 mA, induces an body sway to the anodal side, i.e. to the left, in bipedal standing (Day, 1999; Lund and Broberg, 1983).

### Unilateral muscle vibration

Two muscle vibrators, consisting of DC motors (Maxon International AG, Sachseln, Switzerland) driving an eccentric mass in a PVC case, were placed 2 cm lateral to the L3 spinous processes on both the left and right paraspinal muscles. Vibrators were fixed tightly with double-sided tape and an elastic band. Only the right vibrator was activated (80 Hz, with an amplitude of approximately 2 mm), which induces an illusion of right paraspinal muscle lengthening (Courtine et al., 2007).

### Kinematics and ground reaction force

Whole-body kinematics were recorded using a 3D motion capture system (Optotrak, Northern Digital Inc., Waterloo, ON, Canada) sampling at 50 samples s^−1^ with cluster markers on the feet, shanks, thighs, pelvis, trunk, head, upper arms and forearms. Corresponding anatomical landmarks were digitized with a six-marker probe based on the model described by Kingma et al. (1996). Ground reaction forces were measured at 1000 samples s^−1^ by force plates embedded in the split-belt treadmill (ForceLink b.v., Culemborg, The Netherlands).

### Protocol

For familiarization, participants were exposed to 5-s step-like EVS and muscle vibration during unipedal standing, with three repetitions of each stimulation. Participants then walked on the dual-belt treadmill at the fastest (5.0 km h^−1^) and slowest selected speed (0.8 km h^−1^) for 2 min at each speed without any stimulation. The subsequent conditions were defined by the stimulation received (EVS or muscle vibration) and by postural tasks (unipedal standing, bipedal standing, sitting, and walking at five different speeds). The order of conditions was randomized. All conditions were performed with eyes open. With the configurations in this study, EVS typically induces a compensatory body sway to the left, whereas unilateral muscle vibration typically induces a compensatory body sway to the right during bipedal standing.

During unipedal standing, participants stood on their right foot with arms relaxed at their sides. Trials were repeated if the non-stance leg touched the ground. For bipedal standing, participants stood with their feet shoulder-width apart and arms relaxed at their sides, whereas for sitting, participants sat upright on a stool with arms crossed at shoulder height, feet on the ground and knees flexed at 90 deg. In all postural conditions, participants were instructed to minimize the movement. The 5-s stimulation (muscle vibration or EVS) in standing and sitting trials was manually triggered by the researcher with approximately 10-s intervals before and after stimuli, which aimed to ensure that the participant could not anticipate the stimulus. Each postural task included 10 repetitions of each stimulation. Each repetition lasted 25 s in total.

For the walking conditions, participants walked on the dual-belt treadmill at speeds of 0.8, 2.0, 3.2, 4.3 and 5.5 km h^−1^ for 4 min at each speed. For both EVS and muscle vibration, the stimulation was automatically triggered at right heel strikes and lasted 5 s, with approximately 10-s intervals between stimulations.

### Data analysis

Ground reaction forces were downsampled to 50 Hz. Gait events were identified based on the combined centre of pressure (Roerdink et al., 2008). The mediolateral displacement of markers placed on the T6 spinal process and sacrum were analysed to assess the response to stimulations. A negative mediolateral displacement indicated a leftward movement.

**Fig. 1.**
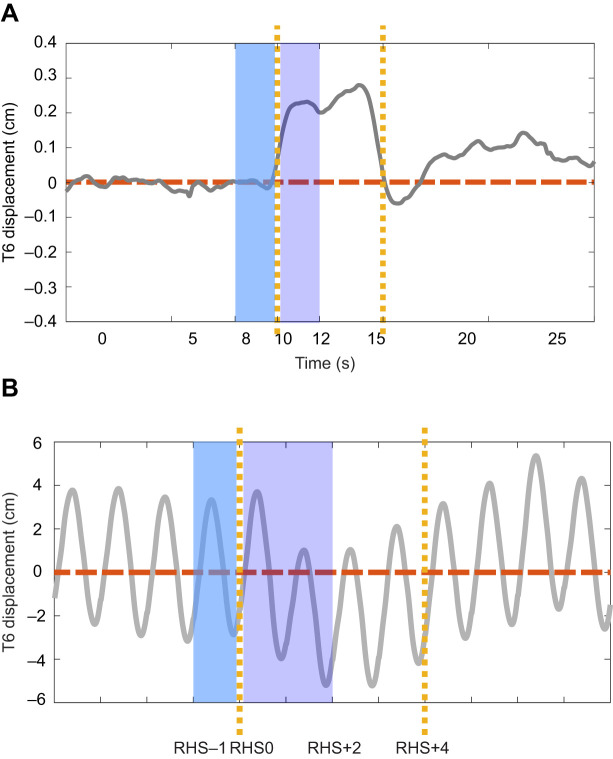
**Example of signal analysis.** Example of signal analysis of data collected during (A) bipedal standing and (B) walking at 3.2 km h^−1^. Grey lines represent the mean T6 trajectory at the group level, averaged across repetitions and participants after offset removal. The vertical yellow dashed lines indicate the onset and offset of muscle vibration. Shaded areas represent the baselines (blue) and responses (purple) used for averaging. RHS, right heel strike.

To calculate marker displacement, the trajectories of the T6 and sacrum markers were filtered using a bi-directional fourth-order, 10 Hz Butterworth low pass filter. For the walking conditions, the marker trajectories were normalized to the gait cycle starting from right heel strikes. To remove the offset, the baseline for each repetition, defined as the mean marker position over 2 s before the onset of perturbation in standing and sitting, and over one stride before the onset of perturbation in walking, was subtracted from the marker trajectory for each repetition, trial and participant. Marker displacement was then calculated as the mean position during the first 2 s after the trigger in standing and sitting tasks, and the first two strides after the trigger in walking. The window size was chosen based on a previous study showing that no turning was induced by EVS during overground walking until the second step, and the initial turn (up to 3 steps) lasted for 2.3 to 3.5 s (Fitzpatrick et al., 1999). We also analyzed 1-s and 3-s windows in standing and sitting. It confirmed that the main results were consistent, except for larger effect of perturbations in unipedal standing with the 3-s window, which may be due to gravitational effects from compensatory movement. The average displacement was calculated across repetitions for each participant, and then across participants (Fig. 1). Anteroposterior (AP) displacement of the T6 markers were also analyzed. The standard deviations of T6 displacement in the AP direction were calculated for baselines and perturbed gait cycles at all walking speeds.

### Statistical analysis

To verify that EVS and muscle vibration predominantly induced perturbations in the mediolateral direction during walking, one-sample *t*-tests were performed comparing changes in the standard deviation of T6 displacement in the AP direction to zero. To evaluate whether there were effects of EVS and muscle vibration on trunk stabilization, one-sample *t*-tests were performed to compare the mediolateral displacements for each condition to zero. To assess whether the effect of EVS and muscle vibration varied across postures in standing and sitting or speed in walking tasks, we used one-way repeated-measures ANOVAs with the factors postural task and walking speed. Mauchly's test was used to check the assumption of sphericity, and Greenhouse–Geisser corrections were applied when the assumption was rejected. Non-integer values in degrees of freedom were a result of Greenhouse–Geisser correction. A Bonferroni correction was applied for the *post hoc* tests when appropriate. A *P*-value less than 0.05 was defined as significant. Effect size was estimated by Cohen's *d* for *t*-tests and partial eta-squared (η_p_^2^) for ANOVA tests. Statistical analyses were performed in MATLAB (2019a, MathWorks, Natick, MA, USA).

## RESULTS

### Standing and sitting

**Fig. 2.**
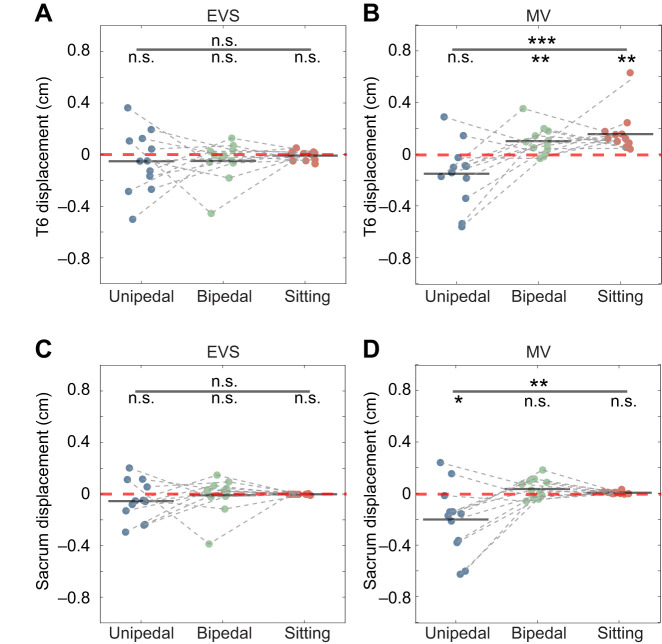
**Response during standing and sitting.** Mediolateral displacement of T6 (A,B) and the sacrum (C,D) in response to electrical vestibular stimulation (EVS; A,C) and muscle vibration (MV; B,D) during unipedal standing (blue dots), bipedal standing (green dots) and sitting (red dots) (*N*=13 for all conditions). Negative values (in cm) indicate leftward displacements. Group-level displacements are shown as black horizontal bars, and individual displacements are represented by filled dots. MV induced significant displacement of T6 during bipedal standing (*P*=0.008) and sitting (*P*=0.003), and significant displacement of the sacrum during unipedal standing (*P*=0.023; one-sample *t*-test against zero). No significant responses were induced by EVS in any condition. Repeated-measures ANOVAs showed that postural task had no significant effect on EVS-induced displacement but significantly affected MV-induced displacement of T6 (*F*_1.686,18.546_=10.655, *P*<0.001) and the sacrum (*F*_2,22_=8.063, *P*=0.002). Asterisks indicate significant *P*-value (*P<0.05, **P<0.01, ***P<0.001), and n.s. indicates non-significance.

EVS induced no significant mediolateral displacement of T6 during unipedal standing (mean±s.d.: −0.05±0.24 cm, *P*=0.469, *d*=−0.21), bipedal standing (−0.05±0.15 cm, *P*=0.281, *d*=−0.33) or sitting (–0.01±0.36 cm, *P*=0.467, *d*=−0.03) (Table 1). There was no significant effect of postural task on EVS-induced displacement of T6 (*F*_2,22_=0.242, *P*=0.787, η_p_^2^=0.02) (Fig. 2A). Also, no significant mediolateral displacement of the sacrum was induced by EVS during unipedal standing (−0.05±0.16 cm, *P*=0.249, *d*=−0.31), bipedal standing (−0.01±0.14 cm, *P*=0.820, *d*=−0.07) or sitting (−0.00±0.00 cm, *P*=0.06, *d*=0.00) (Table 1). There was no significant effect of postural task on EVS-induced displacement of the sacrum (*F*_2,22_=0.600, *P*_task_=0.588, η_p_^2^=0.05) (Fig. 2B).

**Table 1.** Summary of the one-sample *t*-tests on stimulation-induced mediolateral displacements during unipedal standing, bipedal standing and sitting

Postural task	T6 displacement						Sacrum displacement					
	EVS			MV			EVS			MV		
	Mean±s.d. (cm)	*P*	*d*	Mean±s.d. (cm)	*P*	*d*	Mean±s.d. (cm)	*P*	*d*	Mean±s.d. (cm)	*P*	*d*
Unipedal	−0.05±0.24	0.469	−0.21	−0.15±0.25	0.056	−0.60	−0.05±0.16	0.249	−0.31	−0.20±0.26	0.023	−0.77
Bipedal	−0.05±0.15	0.281	−0.33	0.10±0.11	0.008	0.91	−0.01±0.14	0.820	−0.07	0.03±0.07	0.136	0.43
Sitting	−0.05±0.15	0.467	−0.03	0.16±0.14	<0.001	1.14	−0.00±0.00	0.061	0.00	0.00±0.01	0.069	0.00

EVS, electrical vestibular stimulation; MV, muscle vibration.

Muscle vibration induced a non-significant leftward displacement of T6 during unipedal standing (−0.15±0.25 cm, *P*=0.056, *d*=−0.60) and a significant rightward displacement of T6 in bipedal standing (0.10±0.11 cm, *P*=0.008, *d*=0.91) and sitting (0.16±0.14 cm, *P*<0.001, *d*=1.14) (Table 1). The repeated-measures ANOVA confirmed a significant effect of postural task on the muscle-vibration-induced displacement of T6 (*F*_1.686,18.546_=10.655, *P*<0.001, η_p_^2^=0.49) (Fig. 2A). For the sacrum, muscle vibration induced a significant leftward displacement in unipedal standing (−0.20±0.26 cm, *P*=0.023, *d*=−0.77), but no significant displacement in bipedal standing (0.03±0.07 cm, *P*=0.136, *d*=0.43) or sitting (0.00±0.01 cm, *P*=0.069, *d*=0.00) (Table 1). There was a significant effect of postural task on the muscle-vibration-induced displacement of the sacrum (*F*_2,22_=8.063, *P*=0.002, η_p_^2^=0.42) (Fig. 2B).

### Walking

No significant effect of either EVS or muscle vibration (MV) were found on the T6 displacement in the AP direction during walking at all speeds, confirming that perturbations were induced predominantly in the mediolateral direction during walking (*P*_0.8-EVS_=0.835, *d*=−0.06; *P*_0.8-MV_=0.144, *d*=0.45; *P*_2.0-EVS_=0.189, *d*=−0.40; *P*_2.0-MV_=0.803, *d*=0.07; *P*_3.2-EVS_=0.083, *d*=−0.55; *P*_3.2-MV_=0.608, *d*=−0.15; *P*_4.3-EVS_=0.527, *d*=−0.19; *P*_4.3-MV_=0.706, *d*=−0.11; *P*_5.5-EVS_=0.109, *d*=−0.50; *P*_5.5-MV_=0.787, *d*=−0.08).

EVS and muscle vibration induced significant leftward T6 and sacrum displacements at all walking speeds (Table 2). Speed significantly influenced the displacement at both levels, in both stimulation conditions (EVS–T6: *F*_3.052,33.572_=5.707, *P*<0.001, η_p_^2^=0.34; EVS–sacrum: *F*_2.976,32.736_=4.551, *P*=0.004, η_p_^2^=0.29; MV–T6: *F*_3.068,33.748_=6.304, *P*=0.002, η_p_^2^=0.36; MV–sacrum: *F*_3.412,37.532_=4.870, *P*=0.002, η_p_^2^=0.41).

**Table 2.** Summary of the one-sample *t*-tests on stimulation-induced mediolateral displacements during walking

Speed (km h^−1^)	T6 displacement						Sacrum displacement					
	EVS			MV			EVS			MV		
	Mean±s.d. (cm)	*P*	*d*	Mean±s.d. (cm)	*P*	*d*	Mean±s.d. (cm)	*P*	*d*	Mean±s.d. (cm)	*P*	*d*
0.8	−0.41±0.49	0.016	−0.84	−0.41±0.56	0.030	−0.73	−0.28±0.38	0.024	−0.74	−0.37±0.49	0.023	−0.76
2.0	−1.56±1.37	0.002	−1.14	−0.89±0.46	<0.001	−1.93	−1.07±1.09	0.006	−0.98	−0.72±0.39	<0.001	−1.85
3.2	−1.16±1.30	0.010	−0.89	−1.61±0.56	<0.001	−2.88	−0.92±1.12	0.015	−0.82	−1.00±0.63	<0.001	−1.59
4.3	−1.38±0.90	<0.001	−1.53	−0.98±0.57	<0.001	−1.72	−1.12±0.81	<0.001	−1.38	−0.84±0.57	<0.001	−1.47
5.5	−1.09±0.92	0.001	−1.18	−0.44±0.53	0.016	−0.83	−0.77±0.71	0.003	−1.08	−0.34±0.55	0.050	−0.62

**Fig. 3.**
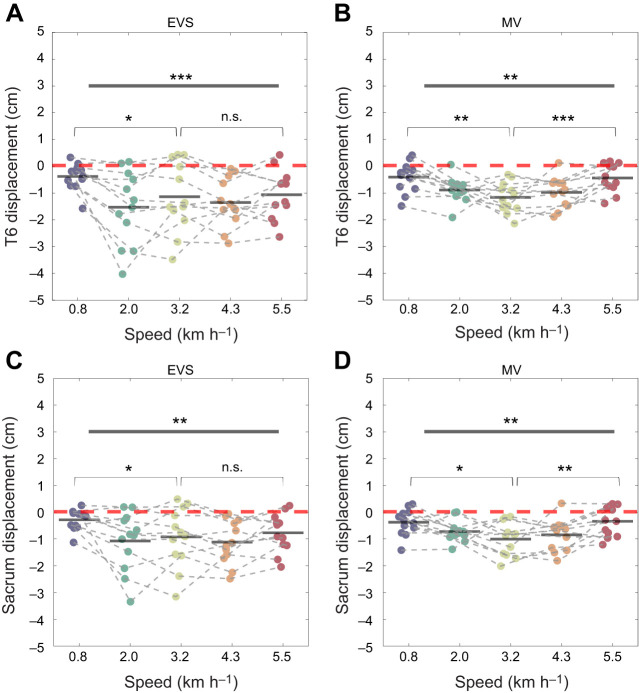
**Responses during walking.** Mediolateral displacement of T6 (A,B) and the sacrum (C,D) in response to electrical vestibular stimulation (EVS; A,C) and muscle vibration (MV; B,D) during walking at five different speeds (*N*=13 for all conditions). Negative values (in cm) indicate leftward displacements. Group-level displacements are shown as black horizontal bars, and individual displacements are represented by filled dots. Significant effects of speed were found on displacement in response to EVS at both the T6 and sacrum levels (repeated-measures ANOVAs). In the MV conditions, induced responses also varied significantly with speed at T6 and the sacrum. *Post hoc* tests indicated that responses at the slowest (0.8 km h^−1^) speed were significantly smaller than those at the moderate speed (3.2 km h^−1^) in all conditions (paired *t*-test). Responses at the highest speed (5.5 km h^−1^) were also significantly smaller than at than those at the moderate speed (3.2 km h^−1^) in the MV conditions, but not in the EVS conditions. Asterisks indicate significant *P*-value (*P<0.05, **P<0.01, ***P<0.001), and n.s. indicates non-significance.

The speed-dependent responses to stimulations (EVS and muscle vibration) at both T6 and sacrum levels appeared to follow a non-monotonic pattern. Specifically, the induced mediolateral displacement increased from slow speeds (e.g. from 0.8 to 2.0 km h^−1^), peaked at a moderate speed (around 3.2 km h^−1^) and then decreased at higher speeds (approximately from 4.3 to 5.5 km h^−1^) (Fig. 3). To confirm the non-linearity, *post hoc* tests comparing the slowest (0.8 km h^−1^) and fastest speed (5.5 km h^−1^) to the moderate speed (3.2 km h^−1^) were performed with paired *t*-tests.

Responses to muscle vibration at both T6 and the sacrum significantly increased from the slowest to moderate speed (T6: *P*=0.004, *d*=1.04; sacrum: *P*=0.017, *d*=0.81) and then decreased at the highest speed (T6: *P*<0.001, *d*=−1.31; sacrum: *P*=0.005, *d*=−0.99), consistent with a U-shaped pattern.

Responses to EVS at T6 and the sacrum significantly increased from the slowest to the moderate speed (T6: *P*=0.032, *d*=0.71; sacrum: *P*=0.045, *d*=0.65), whereas no significant differences were found between the moderate and highest speed (T6: *P*=0.704, *d*=−0.11; sacrum: *P*=0.402, *d*=−0.25). Therefore, EVS-induced responses increased from very slow to moderate walking speeds and plateaued at higher speeds.

## DISCUSSION

We investigated the task-specific contribution of the vestibular and lumbar proprioceptive systems to trunk stabilization during sitting, unipedal and bipedal standing, and walking at five different speeds. Contrary to our hypothesis, EVS did not evoke significant mediolateral displacements during sitting, bipedal standing or unipedal standing. Muscle vibration evoked task-dependent responses, i.e. it induced a significant leftward displacement of the sacrum during unipedal standing, whereas evoking a significant rightward displacement at T6 only during bipedal standing and sitting. During walking, both EVS and muscle vibration consistently induced significant leftward displacements of T6 and the sacrum across all speeds. In line with our hypothesis, the magnitude of these responses varied with walking speed. Below, we discuss how these findings advance our understanding of the task-specific contribution of the vestibular and lumbar proprioceptive systems to the trunk stabilization.

### EVS-induced responses during standing and sitting

No significant responses to EVS at the T6 and sacrum levels were found during sitting, unipedal standing or bipedal standing. This contrasts with previous studies, which observed a significant displacement of centre of pressure, head, torso and pelvis towards the anodal side in response to EVS during bipedal standing (Day et al., 1997; Fitzpatrick et al., 1999; Lund and Broberg, 1983). Our null effect could be explained by the availability of visual information. Previous studies were performed with eyes closed. Hence, in such a condition, vestibular input was likely upweighted, increasing the effect of EVS. Consistent with this, EVS-induced motor responses during bipedal standing were smaller with eyes open compared with closed (Britton et al., 1993; Fitzpatrick and Day, 2004). Furthermore, a study showed that during bipedal standing on a stable surface, reliance on ankle proprioception was greater than on vestibular signals, and this reliance increased with the presence of EVS (Cenciarini and Peterka, 2006). Therefore, even though our sample size and stimulation intensity were comparable to those in previous studies with eyes closed (Day et al., 1997; Fitzpatrick et al., 1999; Lund and Broberg, 1983), the intensity of EVS may not have been sufficient to evoke a significant effect on trunk stabilization during unipedal and bipedal standing owing to sensory reweighting.

However, another study reported that the presence of vision did not significantly modulate the EVS-induced lumbar muscle responses during sitting (Amélie et al., 2021). The authors attributed this to the strong tactile input from the thighs and buttocks during sitting. Reweighting between vestibular and tactile information has been confirmed by a reduced coupling between EVS and the centre of pressure trajectory during bipedal standing when providing fingertip contact (Goar et al., 2025). From a biomechanical point of view, the large contact of the thighs and pelvis with the seat provides a larger base of support compared with standing. The height of the centre of mass relative to the base of support is lower during sitting; consequently, a smaller destabilizing torque will be generated when the trunk has a given angular deviation from the upright posture compared to standing. For these reasons, the gain of responses to EVS induced illusions is likely to be smaller during sitting than during unipedal and bipedal standing.

### EVS-induced responses during walking

During walking, we found that at both the T6 and sacrum levels, EVS induced a significant displacement to the left, i.e. the anodal side, in line with previous literature (Bent et al., 2004; Fitzpatrick et al., 1999; Jahn et al., 2000; Iles et al., 2007). The vestibular signal encodes the head orientation in space (Cullen, 2012), and this perceived vestibular signal needs to be integrated with neck proprioception to distinguish whether the whole body is moving or only the head is moving on the stationary trunk (Pettorossi and Schieppati, 2014). In our case, participants were walking facing forward, thus head orientation was on average aligned with the trunk. Consequently, EVS induced an illusion of whole-body movement to the right. A global compensatory response to the left was induced to stabilize the trunk in the upright orientation in space.

The differences in the effects of EVS between standing and walking were partly supported by Fitzpatrick et al. (1999), who reported that EVS-induced body sway during standing was smaller and more variable than during walking. During walking, a stereotyped and symmetric locomotion, the contribution of vestibular signals is phasic (Blouin et al., 2011; Ivanenko et al., 2000; Li et al., 2024; O'Connor and Kuo, 2009). As such, the phase-locked step-like EVS-induced perturbation is likely to be consistent over strides. This phasic vestibular contribution has also been reported in non-human animals, with enhanced responses to vestibular stimulation at the initial stance phase, presumably to provide antigravity support and maintain the equilibrium of the trunk (Marlinsky, 1992). Biomechanically, vestibular signals are selectively used during phases where stabilization demands are greatest owing to changes in the base of support (Fitzpatrick et al., 1999; Magnani et al., 2021). In contrast, during standing and sitting, the responses to EVS can superimpose negatively or positively on the ongoing body sway, which would increase the variability in measured responses. In addition, the larger and more consistent responses also suggest increased weighting of vestibular signal, likely reflecting its role in controlling heading direction during walking. It has been shown that, compared with quiet standing, EVS-induced responses increased when subjects voluntarily tilted their bodies laterally or forward while standing (Cauquil and Day, 1998; Smetanin et al., 1988). This increase was attributed to the contribution of vestibular signals to spatial perception during voluntary movements (Cauquil and Day, 1998).

### Muscle-vibration-induced responses during standing and sitting

**Fig. 4.**
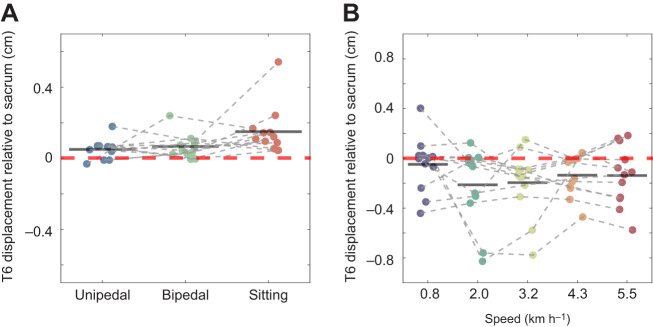
**MV-induced T6–sacrum relative displacement.** MV-induced relative displacement between T6 and the sacrum in the mediolateral direction across tasks: (A) during unipedal standing (blue dots), bipedal standing (green dots) and sitting (red dots); and (B) during walking at five different speeds. Negative values indicate leftward displacement of T6 relative to the sacrum (in cm). Group-level displacements are shown as black horizontal bars, and individual displacements are represented by filled dots.

Muscle vibration causes an illusion of lengthening of the vibrated muscle (Goodwin et al., 1972). Consistent with the literature, during bipedal standing and sitting, a significant rightward T6 displacement was found (Courtine et al., 2007). No significant sacrum displacement was found in these tasks. In contrast, during unipedal standing, a sacrum displacement, but no trunk displacement, was observed. Therefore, we analysed the relative position of T6 to the sacrum (Fig. 4A). A rightward displacement of T6 relative to the sacrum during unipedal standing was found in all three postures. Thus, the different response during unipedal standing versus sitting and bipedal standing could be caused by a difference in the interpretation of the postural change related to trunk muscle lengthening. During bipedal standing and sitting, the sacrum is likely to remain stable, given the wide and symmetrical support. Thus, muscle lengthening is likely to be interpreted as a leftward trunk movement relative to the sacrum, inducing a compensatory rightward trunk movement. However, in unipedal standing with a less stable and asymmetric support of the pelvis, this perceived lengthening is possibly to be interpreted as a rightward displacement of the pelvis, inducing a leftward sacrum displacement to re-align the segments.

### Muscle-vibration-induced responses during walking

Similar to unipedal standing, during walking, the vibration-induced perception of paraspinal muscle lengthening may be interpreted as a pelvis movement. With vibration on the hip abductor muscle of the stance leg, an inward placement of the contralateral foot was reported, which suggests that this vibration similarly induced an illusion of pelvis displacement towards the stance side (Arvin et al., 2018). In line with this, we found that lumbar muscle vibration induced a significant leftward displacement at the T6 and sacrum levels opposite to the vibrated side, also consistent with previous studies using trunk muscle vibration (Courtine et al., 2007; Schmid et al., 2005). Unlike during unipedal standing, the T6 displacement was more to the left relative to the sacrum during walking (Fig. 4B). Thus, these responses during walking seem not to be the result of balance control, for which we expected a rightward displacement of T6 relative to the sacrum as found in standing and sitting.

Unilateral lengthening of paraspinal muscles can result not only from trunk or pelvis displacement in the frontal plane, but also from a change in orientation in the transverse plane. Schmid et al. (2005) found that during overground walking with eyes either opened or closed, lumbar paraspinal muscle vibration evoked a deviation in walking trajectory. Additionally, deviations in walking direction were evoked by vibration applied to abdominal muscles and paraspinal muscles at thoracic and lumbar levels, but not with vibrations on lower limb muscles, such as hip abductors and adductors (Courtine et al., 2007). Therefore, trunk proprioceptive information appears to play a major role in the control of balance as well as the control of the heading direction during walking.

Interestingly, muscle vibration induced local responses in static conditions but global responses during walking. This likely reflects that proprioceptive inputs from multiple sources are integrated, whereas the interpretation in terms of an induced illusion depends on the functional role of proprioception in different tasks and postures. This is consistent with a previous study showing that bilateral neck muscle vibration evoked local head movement when the trunk was supported, but induced whole-body sway without trunk support during sitting and standing (Smetanin et al., 1993).

### Effects of walking speed

The effect of EVS and muscle vibration on trunk stabilization varied with walking speed. Contrary to our hypotheses, in the selected range of speeds from 0.8 to 5.5 km h^−1^, responses to both stimuli increased from very slow speeds to moderate speeds. This may be in line with the phenomenon that specific patient groups with sensory loss or dysfunction tend to walk slower (Hicks et al., 2017; Menz et al., 2004; Wuehr et al., 2014). Some studies suggested that slow walking is more stable than fast walking (Dingwell and Marin, 2006; England and Granata, 2007). However, this conclusion has been debated (Best and Wu, 2020; Bruijn et al., 2009; Hak et al., 2012).

Smaller feedback responses at very low speeds may be a result of the increased duration of the stance phases, especially of the double support phases, both in absolute sense and as a percentage of the gait cycle (Wu et al., 2019; Wuehr et al., 2014). Given the long duration of the double support phases in very slow walking (e.g. 0.8 km h^−1^), the control strategy may resemble that during standing, where we found smaller or no effects of sensory stimulation. However, previously we found that trunk muscle modulation in response to vestibular afference mainly took place during the double support phase (Li et al., 2024).

Another possible explanation for the smaller feedback responses at very low speeds is that walking at such slow speed is controlled as a repeated transition between standing and walking. The optimal feedback control theory suggests that to enable the transition from one posture or task to another, the control set for the preceding needs to be disengaged before setting sensory feedback gains for the subsequent task (Cluff and Scott, 2016). This prediction was supported by findings from Tisserand et al. (2018), who reported absence of coupling between EVS and ground reaction force during the transition between standing and walking. Hence, when very slow walking is regarded as a repeated transition between standing and walking, the overall feedback gains should be low.

From moderate to higher walking speeds, responses to EVS plateaued rather than decreased, in contrast to previous reports of reduced EVS effects at higher locomotion speeds (Brandt et al., 1999; Dakin et al., 2013; Fabre-Adinolfi et al., 2018; Jahn et al., 2000). This discrepancy may be partly explained by differences in experimental design, as most of these studies compared walking with running (Brandt et al., 1999; Fabre-Adinolfi et al., 2018; Jahn et al., 2000). Biomechanically, running differs from fast walking by the absence of a double support phase, during which vestibular afference mostly contributes to trunk muscle modulation during walking (Li et al., 2024). Therefore, reduced EVS effects observed during running compared with walking may reflect changes in gait mechanics across the transition between walking and running rather than a monotonic effect in vestibular contribution with increasing speeds in walking.

Dakin et al. (2013) found reduced EVS-evoked responses in lower limb muscles when walking at 0.8 m s^−1^ (2.9 km h^−1^) compared with 0.4 m s^−1^ (1.4 km h^−1^). These speeds fall within the interval between speed conditions in the present study (0.8, 2.0 and 3.2 km h^−1^). Moreover, they applied stochastic EVS signals rather than the step-like EVS. These methodological differences may also contribute to the different findings between the present and previous studies. In addition, our statistical power could be limited as there was a small effect size and we had a limited sample size. Therefore, a slight reduction in responses to EVS at higher speed cannot be excluded based on the current data.

Based on previous studies of reduced effects of vestibular and visual perturbations on walking stabilization at higher walking speed (Brandt et al., 1999; Jahn et al., 2000, 2001; Dakin et al., 2013; Fabre-Adinolfi et al., 2018), we expected that the proprioceptive signal would compensate during fast walking, reflected by a larger muscle-vibration-induced response expected with increased walking speed. However, response to muscle vibration decreased from moderate to higher speeds. This finding is consistent with the reduced activity of the vestibular and somatosensory cortex during imagined running compared with imagined slow speed walking and standing (Jahn et al., 2004), suggesting a decreased reliance on feedback control at higher speeds. This interpretation can be further supported by a recent study showing a reduced energetic cost of walking stabilization at fast walking compared with a slow walking (Muijres et al., 2026).

It is believed that stable walking is achieved by the interaction of multisensory feedback control, automated central pattern generators, and modulation of intrinsic mechanical properties of the body (Grillner and El Manira, 2020; MacKay-Lyons, 2002; Wuehr et al., 2013). In animal models without movement-related sensory feedback, automated central pattern generators can generate the rhythmic muscle activity for locomotion (Dubuc et al., 1986; Grillner et al., 1976; Lambert et al., 2012). It is therefore plausible that in humans at faster walking speeds where locomotion becomes more rhythmic, the automated locomotor control partially replaces feedback control. Additionally, the decreased effects of muscle vibration at higher speeds may also be explained by biomechanical factors. At faster walking speeds, trunk muscle activity is increased (Anders et al., 2007), which would result in increased muscle stiffness and, consequently, increased trunk stiffness.

### Limitations

In this study, we did not statistically compare the contribution of vestibular and proprioceptive information between the static and dynamic tasks for several reasons. Dependent on the task, sensory information may be used not just for balance control. As we argue above, trunk proprioceptive information also contributes to the control of walking direction. Moreover, the responses to stimulation were analysed differently between walking and static conditions. Additionally, differences in trunk control strategies could further complicate comparisons between postures and tasks. For example, the responses to muscle vibration at the trunk and pelvis level differ between unipedal standing, bipedal standing and sitting. In addition, the demands on trunk control differ between postures. For example, trunk deviations generate smaller destabilizing torques during sitting than during standing because the centre of mass is located lower relative to the base of support. Together, these factors could confound the comparison of response magnitudes to stimulation, i.e. the gain of feedback response, between static and dynamic tasks.

Another limitation is that we cannot investigate whether the relative weights of the vestibular and proprioceptive signals differed between tasks and postures for trunk stabilization. We found parallel trends in the task- and speed-related modulation of responses evoked by EVS and muscle vibration, which possibly indicates an overall modification of the feedback control gain. At the same time, reweighting between vestibular and proprioceptive signals could also exist for optimal feedback control in different task and postures. However, experimentally quantifying sensory weighting is challenging. Although EVS and muscle vibration provide direct perturbations to the vestibular system and proprioception, the resulting balance responses likely represent integrated multisensory feedback, in which sensory reweighting due to sensory perturbations may already have occurred. Combining experimental approaches with computational modelling provides a powerful tool to quantify changes in sensory weights in unperturbed conditions. Within neuromusculoskeletal models, each sensory modality can be independently adjusted by changing parameters such as noise amplitudes, and the effects on balance performance can be evaluated with simulations (Wouwe et al., 2022).

We performed two separate repeated-measures ANOVAs for the T6 and sacrum markers to evaluate the effects of EVS or muscle vibration across postural tasks. This may increase the risk of Type I errors by testing the datasets from T6 and the sacrum independently, as biomechanically, the motions of T6 and the sacrum are not independent. Considering the marker position as a factor in the MANOVA test could be a reasonable solution. However, if a significant effect of marker position would be found, ANOVAs for each marker should be performed as *post hoc* tests. In this case, the main finding should remain the same as current findings, i.e. the contribution of vestibular and proprioceptive information in trunk stabilization differs between postural tasks and walking speeds.

To test our hypotheses, we averaged marker displacements across repetitions to extract the consistent directional responses to muscle vibration and EVS across postures and tasks. However, averaging does eliminate temporal information on the responses. With the current dataset, future work could access how responses change across repetitions to provide insight into potential adaptation and dynamic reweighting of sensory inputs.

### Conclusions

In conclusion, the present findings confirm that the contribution of vestibular and proprioceptive afference to trunk stabilization varies between postures and walking speeds. Muscle vibration induced different responses at the T6 and sacrum levels in unipedal standing compared with sitting and bipedal standing. This suggests a different interpretation of the sensation of muscle lengthening in these postures. By extending the range of selected walking speeds compared with previous studies, we found decreased responses to stimulation at very slow speeds. This suggests that a different control strategy is used during slow walking, possibly owing to the long double stance duration, or perhaps because very slow walking reflects a repeated initiation of walking rather than a steady-state process. From moderate to higher speeds, the response to EVS plateaued, which could be due to methodological differences to literatures and limited effect size in the present study. Therefore, a slight reduction in responses to EVS at higher speed cannot be excluded. However, responses to muscle vibration significantly decreased. Overall, these findings suggest that stabilization of faster walking relies less on feedback control.

## References

[JEB251082C1] Alberts, B., Selen, L. P. J. and Medendorp, W. P. (2019). Age-related reweighting of visual and vestibular cues for vertical perception. *J. Neurophysiol.* 121, 1279-1288. 10.1152/jn.00481.2018 30699005 PMC6485738

[JEB251082C2] Ali, A. S., Rowen, K. A. and Iles, J. F. (2003). Vestibular actions on back and lower limb muscles during postural tasks in man. *J. Physiol.* 546, 615-624. 10.1113/jphysiol.2002.030031 12527747 PMC2342524

[JEB251082C3] Amélie, D., Mikaël, D., Jean-Philippe, C., Martin, S. and Hugo, M.-A. (2021). Motor responses of lumbar erector spinae induced by electrical vestibular stimulation in seated participants. *Front. Hum. Neurosci.* 15, 690433. 10.3389/fnhum.2021.690433 34366814 PMC8339290

[JEB251082C4] Anders, C., Wagner, H., Puta, C., Grassme, R., Petrovitch, A. and Scholle, H.-C. (2007). Trunk muscle activation patterns during walking at different speeds. *J. Electromyogr. Kinesiol.* 17, 245-252. 10.1016/j.jelekin.2006.01.002 16517182

[JEB251082C5] Anson, E., Agada, P., Kiemel, T., Ivanenko, Y., Lacquaniti, F. and Jeka, J. (2014). Visual control of trunk translation and orientation during locomotion. *Exp. Brain. Res.* 232, 1941-1951. 10.1007/s00221-014-3885-1 24658632 PMC4087056

[JEB251082C6] Arvin, M., Hoozemans, M. J. M., Pijnappels, M., Duysens, J., Verschueren, S. M. and van Dieën, J. H. (2018). Where to step? Contributions of stance leg muscle spindle afference to planning of mediolateral foot placement for balance control in young and old adults. *Front. Physiol.* 9, 1134. 10.3389/fphys.2018.01134 30246780 PMC6110888

[JEB251082C7] Asslander, L. and Peterka, R. J. (2014). Sensory reweighting dynamics in human postural control. *J. Neurophysiol.* 111, 1852-1864. 10.1152/jn.00669.2013 24501263 PMC4044370

[JEB251082C8] Bent, L. R., McFadyen, B. J. and Inglis, J. T. (2004). Is the use of vestibular information weighted differently across the initiation of walking? *Exp. Brain. Res.* 157, 407-416. 10.1007/s00221-004-1854-9 14991215

[JEB251082C9] Best, A. N. and Wu, A. R. (2020). Upper body and ankle strategies compensate for reduced lateral stability at very slow walking speeds. *Proc. Biol. Sci.* 287, 20201685. 10.1098/rspb.2020.1685 33049173 PMC7657866

[JEB251082C10] Blouin, J. S., Dakin, C. J., van den Doel, K., Chua, R., McFadyen, B. J. and Inglis, J. T. (2011). Extracting phase-dependent human vestibular reflexes during locomotion using both time and frequency correlation approaches. *J. Appl. Physiol.* 111, 1484-1490. 10.1152/japplphysiol.00621.2011 21868684

[JEB251082C11] Brandt, T., Strupp, M. and Benson, J. (1999). You are better off running than walking with acute vestibulopathy. *Lancet* 354, 746. 10.1016/S0140-6736(99)03179-7 10475195

[JEB251082C12] Britton, T. C., Day, B. L., Brown, P., Rothwell, J. C., Thompson, P. D. and Marsden, C. D. (1993). Postural electromyographic responses in the arm and leg following galvanic vestibular stimulation in man. *Exp. Brain Res.* 94, 143-151. 10.1007/BF00230477 8335069

[JEB251082C13] Bruijn, S. M., van Dieën, J. H., Meijer, O. G. and Beek, P. J. (2009). Is slow walking more stable? *J. Biomech.* 42, 1506-1512. 10.1016/j.jbiomech.2009.03.047 19446294

[JEB251082C14] Cauquil, A. S. and Day, B. L. (1998). Galvanic vestibular stimulation modulates voluntary movement of the human upper body. *J. Physiol.* 513, 611-619. 10.1111/j.1469-7793.1998.611bb.x 9807008 PMC2231296

[JEB251082C15] Cenciarini, M. and Peterka, R. J. (2006). Stimulus-dependent changes in the vestibular contribution to human postural control. *J. Neurophysiol.* 95, 2733-2750. 10.1152/jn.00856.2004 16467429

[JEB251082C16] Cluff, T. and Scott, S. H. (2016). Online corrections are faster because movement initiation must disengage postural control. *Mot. Control* 20, 162-170. 10.1123/mc.2015-0027 25920075

[JEB251082C17] Courtine, G., De Nunzio, A. M., Schmid, M., Beretta, M. V. and Schieppati, M. (2007). Stance- and locomotion-dependent processing of vibration-induced proprioceptive inflow from multiple muscles in humans. *J. Neurophysiol.* 97, 772-779. 10.1152/jn.00764.2006 17065250

[JEB251082C18] Cullen, K. E. (2012). The vestibular system: multimodal integration and encoding of self-motion for motor control. *Trends Neurosci.* 35, 185-196. 10.1016/j.tins.2011.12.001 22245372 PMC4000483

[JEB251082C19] Dakin, C. J., Inglis, J. T., Chua, R. and Blouin, J. S. (2013). Muscle-specific modulation of vestibular reflexes with increased locomotor velocity and cadence. *J. Neurophysiol.* 110, 86-94. 10.1152/jn.00843.2012 23576695

[JEB251082C20] Day, B. L. (1999). Galvanic vestibular stimulation: new uses for an old tool. *J. Physiol.* 517, 631. 10.1111/j.1469-7793.1999.0631s.x 10358104 PMC2269367

[JEB251082C21] Day, B. L., Séverac Cauquil, A., Bartolomei, L., Pastor, M. A. and Lyon, I. N. (1997). Human body-segment tilts induced by galvanic stimulation: a vestibularly driven balance protection mechanism. *J. Physiol.* 500, 661-672. 10.1113/jphysiol.1997.sp022051 9161984 PMC1159417

[JEB251082C22] de Leva, P. (1996). Adjustments to Zatsiorsky-Seluyanov's segment inertia parameters. *J. Biomech.* 29, 1223-1230. 10.1016/0021-9290(95)00178-6 8872282

[JEB251082C23] Dingwell, J. B. and Marin, L. C. (2006). Kinematic variability and local dynamic stability of upper body motions when walking at different speeds. *J. Biomech.* 39, 444-452. 10.1016/j.jbiomech.2004.12.014 16389084

[JEB251082C24] Dubuc, R., Rossignol, S. and Lamarre, Y. (1986). The effects of 4-aminopyridine on the spinal cord: rhythmic discharges recorded from the peripheral nerves. *Brain Res.* 369, 243-259. 10.1016/0006-8993(86)90533-0 3008935

[JEB251082C25] Duclos, N. C., Maynard, L., Barthelemy, J. and Mesure, S. (2014). Postural stabilization during bilateral and unilateral vibration of ankle muscles in the sagittal and frontal planes. *J. Neuroeng. Rehabil.* 11, 130. 10.1186/1743-0003-11-130 25178183 PMC4162932

[JEB251082C26] England, S. A. and Granata, K. P. (2007). The influence of gait speed on local dynamic stability of walking. *Gait Posture* 25, 172-178. 10.1016/j.gaitpost.2006.03.003 16621565 PMC1785331

[JEB251082C27] Fabre-Adinolfi, D., Parietti-Winkler, C., Pierret, J., Lassalle-Kinic, B. and Frère, J. (2018). You are better off running than walking revisited: does an acute vestibular imbalance affect muscle synergies? *Hum. Mov. Sci.* 62, 150-160. 10.1016/j.humov.2018.10.010 30384183

[JEB251082C28] Fitzpatrick, R. C. and Day, B. L. (2004). Probing the human vestibular system with galvanic stimulation. *J. Appl. Physiol.* 96, 2301-2316. 10.1152/japplphysiol.00008.2004 15133017

[JEB251082C29] Fitzpatrick, R., Burke, D. and Gandevia, S. C. (1994). Task-dependent reflex responses and movement illusions evoked by galvanic vestibular stimulation in standing humans. *J. Physiol.* 478, 363-372. 10.1113/jphysiol.1994.sp020257 7965852 PMC1155693

[JEB251082C30] Fitzpatrick, R. C., Wardman, D. L. and Taylor, J. L. (1999). Effects of galvanic vestibular stimulation during human walking. *J. Physiol.* 517, 931-939. 10.1111/j.1469-7793.1999.0931s.x 10358131 PMC2269389

[JEB251082C31] Gibson, J. J. (1958). Visually controlled locomotion and visual orientation in animals. *Br. J. Psychol.* 49, 182-194. 10.1111/j.2044-8295.1958.tb00656.x 13572790

[JEB251082C32] Goar, M. H., Barnett-Cowan, M. and Horslen, B. C. (2025). Light touch alters vestibular-evoked balance responses: insights into dynamics of sensorimotor reweighting. *J. Neurophysiol.* 133, 142-161. 10.1152/jn.00166.2024 39625307

[JEB251082C33] Goodwin, G. M., McCloskey, D. I. and Matthews, P. B. C. (1972). Proprioceptive illusions induced by muscle vibration: contribution by muscle spindles to perception? *Science* 175, 1382-1384. 10.1126/science.175.4028.1382 4258209

[JEB251082C34] Grangeon, M., Gauthier, C., Duclos, C., Lemay, J.-F. and Gagnon, D. (2015). Unsupported eyes closed sitting and quiet standing share postural control strategies in healthy individuals. *Mot. Control* 19, 10-24. 10.1123/mc.2013-0091 24718916

[JEB251082C35] Grillner, S. and El Manira, A. (2020). Current principles of motor control, with special reference to vertebrate locomotion. *Physiol. Rev.* 100, 271-320. 10.1152/physrev.00015.2019 31512990

[JEB251082C36] Grillner, S., Perret, C. and Zangger, P. (1976). Central generation of locomotion in the spinal dogfish. *Brain Res.* 109, 255-269. 10.1016/0006-8993(76)90529-1 1276916

[JEB251082C37] Hak, L., Houdijk, H., Steenbrink, F., Mert, A., van der Wurff, P., Beek, P. J. and van Dieën, J. H. (2012). Speeding up or slowing down? Gait adaptations to preserve gait stability in response to balance perturbations. *Gait Posture* 36, 260-264. 10.1016/j.gaitpost.2012.03.005 22464635

[JEB251082C38] Hazime, F. A., Allard, P., Ide, M. R., Siqueira, C. M., Amorim, C. F. and Tanaka, C. (2012). Postural control under visual and proprioceptive perturbations during double and single limb stances: insights for balance training. *J. Bodyw. Mov. Ther.* 16, 224-229. 10.1016/j.jbmt.2011.02.003 22464121

[JEB251082C39] Hicks, G. E., Sions, J. M., Coyle, P. C. and Pohlig, R. T. (2017). Altered spatiotemporal characteristics of gait in older adults with chronic low back pain. *Gait Posture* 55, 172-176. 10.1016/j.gaitpost.2017.04.027 28458149 PMC5493311

[JEB251082C40] Hof, A. L. (2007). The equations of motion for a standing human reveal three mechanisms for balance. *J. Biomech.* 40, 451-457. 10.1016/j.jbiomech.2005.12.016 16530203

[JEB251082C41] Iles, J. F., Baderin, R., Tanner, R. and Simon, A. (2007). Human standing and walking: comparison of the effects of stimulation of the vestibular system. *Exp. Brain. Res.* 178, 151-166. 10.1007/s00221-006-0721-2 17031681

[JEB251082C42] Inglis, J. T., Frank, J. S. and Inglis, B. (1991). The effect of muscle vibration on human position sense during movements controlled by lengthening muscle contraction. *Exp. Brain. Res.* 84, 631-634. 10.1007/BF00230975 1864332

[JEB251082C43] Ivanenko, Y. P., Grasso, R. and Lacquaniti, F. (2000). Influence of leg muscle vibration on human walking. *J. Neurophysiol.* 84, 1737-1747. 10.1152/jn.2000.84.4.1737 11024066

[JEB251082C44] Jahn, K., Strupp, M., Schneider, E., Dieterich, M. and Brandt, T. (2000). Differential effects of vestibular stimulation on walking and running. *Neuroreport* 11, 1745-1748. 10.1097/00001756-200006050-00029 10852236

[JEB251082C45] Jahn, K., Strupp, M., Schneider, E., Dieterich, M. and Brandt, T. (2001). Visually induced gait deviations during different locomotion speeds. *Exp. Brain. Res.* 141, 370-374. 10.1007/s002210100884 11715081

[JEB251082C46] Jahn, K., Deutschländer, A., Stephan, T., Strupp, M., Wiesmann, M. and Brandt, T. (2004). Brain activation patterns during imagined stance and locomotion in functional magnetic resonance imaging. *Neuroimage* 22, 1722-1731. 10.1016/j.neuroimage.2004.05.017 15275928

[JEB251082C47] Kingma, I., de Looze, M. P., Toussaint, H. M., Klijnsma, H. G. and Bruijnen, T. B. M. (1996). Validation of a full body 3-D dynamic linked segment model. *Hum. Mov. Sci.* 15, 833-860. 10.1016/S0167-9457(96)00034-6

[JEB251082C48] Lambert, F. M., Combes, D., Simmers, J. and Straka, H. (2012). Gaze stabilization by efference copy signaling without sensory feedback during vertebrate locomotion. *Curr. Biol.* 22, 1649-1658. 10.1016/j.cub.2012.07.019 22840517

[JEB251082C49] Li, Y. C., Bruijn, S. M., Lemaire, K. K., Brumagne, S. and van Dieën, J. H. (2024). Vertebral level specific modulation of paraspinal muscle activity based on vestibular signals during walking. *J. Physiol.* 602, 507-525. 10.1113/JP285831 38252405

[JEB251082C50] Lund, S. and Broberg, C. (1983). Effects of different head positions on postural sway in man induced by a reproducible vestibular error signal. *Acta Physiol. Scand.* 117, 307-309. 10.1111/j.1748-1716.1983.tb07212.x 6603098

[JEB251082C51] MacKay-Lyons, M. (2002). Central pattern generation of locomotion: a review of the evidence. *Phys. Ther.* 82, 69-83. 10.1093/ptj/82.1.69 11784280

[JEB251082C52] Magnani, R. M., Bruijn, S. M., van Dieen, J. H. and Forbes, P. A. (2021). Stabilization demands of walking modulate the vestibular contributions to gait. *Sci. Rep.* 11, 13736. 10.1038/s41598-021-93037-7 34215780 PMC8253745

[JEB251082C53] Marlinsky, V. V. (1992). Activity of lateral vestibular nucleus neurons during locomotion in the decerebrate guinea pig. *Exp. Brain Res.* 90, 583-588. 10.1007/BF00230942 1426114

[JEB251082C54] Maurer, C., Mergner, T. and Peterka, R. J. (2006). Multisensory control of human upright stance. *Exp. Brain. Res.* 171, 231-250. 10.1007/s00221-005-0256-y 16307252

[JEB251082C55] Mayer, W. P., Murray, A. J., Brenner-Morton, S., Jessell, T. M., Tourtellotte, W. G. and Akay, T. (2018). Role of muscle spindle feedback in regulating muscle activity strength during walking at different speed in mice. *J. Neurophysiol.* 120, 2484-2497. 10.1152/jn.00250.2018 30133381 PMC6295543

[JEB251082C56] Menz, H. B., Lord, S. R., St George, R. and Fitzpatrick, R. C. (2004). Walking stability and sensorimotor function in older people with diabetic peripheral neuropathy1. *Arch. of Phy. Med. Rehabil.* 85, 245-252. 10.1016/j.apmr.2003.06.015 14966709

[JEB251082C57] Muijres, W., Afschrift, M., Ronsse, R. and De Groote, F. (2026). Speeding up, not slowing down, decreases the metabolic energy needed to stabilize walking in the sagittal plane. *J. Appl. Physiol.* 140, 88-97. 10.1152/japplphysiol.00255.2025 41269262

[JEB251082C58] O'Connor, S. M. and Kuo, A. D. (2009). Direction-dependent control of balance during walking and standing. *J. Neurophysiol.* 102, 1411-1419. 10.1152/jn.00131.2009 19553493 PMC2746770

[JEB251082C59] Oie, K. S., Kiemel, T. and Jeka, J. J. (2002). Multisensory fusion: simultaneous re-weighting of vision and touch for the control of human posture. *Cogn. Brain Res.* 14, 164-176. 10.1016/S0926-6410(02)00071-X 12063140

[JEB251082C60] Peterka, R. J. (2002). Sensorimotor integration in human postural control. *J. Neurophysiol.* 88, 1097-1118. 10.1152/jn.2002.88.3.1097 12205132

[JEB251082C61] Peterka, R. J. and Loughlin, P. J. (2004). Dynamic regulation of sensorimotor integration in human postural control. *J. Neurophysiol.* 91, 410-423. 10.1152/jn.00516.2003 13679407

[JEB251082C62] Pettorossi, V. E. and Schieppati, M. (2014). Neck proprioception shapes body orientation and perception of motion. *Front. Hum. Neurosci.* 8, 895. 10.3389/fnhum.2014.00895 25414660 PMC4220123

[JEB251082C63] Prochazka, A. (2021). Proprioception: clinical relevance and neurophysiology. *Curr. Opin. Physiol.* 23, 100440. 10.1016/j.cophys.2021.05.003

[JEB251082C64] Riemann, B. L., Myers, J. B. and Lephart, S. M. (2003). Comparison of the ankle, knee, hip, and trunk corrective action shown during single-leg stance on firm, foam, and multiaxial surfaces. *Arch. Phys. Med. Rehabil.* 84, 90-95. 10.1053/apmr.2003.50004 12589627

[JEB251082C65] Roerdink, M., Coolen, B. H., Clairbois, B. H., Lamoth, C. J. and Beek, P. J. (2008). Online gait event detection using a large force platform embedded in a treadmill. *J. Biomech.* 41, 2628-2632. 10.1016/j.jbiomech.2008.06.023 18657816

[JEB251082C66] Schmid, M., De Nunzio, A. M. and Schieppati, M. (2005). Trunk muscle proprioceptive input assists steering of locomotion. *Neurosci. Lett.* 384, 127-132. 10.1016/j.neulet.2005.04.059 15885899

[JEB251082C67] Schniepp, R., Wuehr, M., Neuhaeusser, M., Kamenova, M., Dimitriadis, K., Klopstock, T., Strupp, M., Brandt, T. and Jahn, K. (2012). Locomotion speed determines gait variability in cerebellar ataxia and vestibular failure. *Mov. Disord.* 27, 125-131. 10.1002/mds.23978 21997342

[JEB251082C68] Smetanin, B. N., Popov, K. E., Gurfinkel, V. S. and Shlykov, V. (1988). Effect of movement and illusion of movement on human vestibulomotor response. *Neurophysiology* 20, 192-196. 10.1007/BF02141337 3260994

[JEB251082C69] Smetanin, B. N., Popov, K. E. and Shlykov, V. (1993). Postural responses to vibrostimulation of the neck muscle proprioceptors in man. *Neurophysiology* 25, 86-92. 10.1007/BF01054155

[JEB251082C70] Takeoka, A., Vollenweider, I., Courtine, G. and Arber, S. (2014). Muscle spindle feedback directs locomotor recovery and circuit reorganization after spinal cord injury. *Cell* 159, 1626-1639. 10.1016/j.cell.2014.11.019 25525880

[JEB251082C71] Tisserand, R., Dakin, C. J., Van der Loos, M. H., Croft, E. A., Inglis, T. J. and Blouin, J. S. (2018). Down regulation of vestibular balance stabilizing mechanisms to enable transition between motor states. *eLife* 7, e36123. 10.7554/eLife.36123 29989550 PMC6056236

[JEB251082C72] Vizirgianakis, S., Amiridis, I. G., Mademli, L., Tsiouri, C. and Hatzitaki, V. (2021). Posture dependent ankle and foot muscle responses evoked by Achilles’ tendon vibration. *Neurosci. Lett.* 759, 135995. 10.1016/j.neulet.2021.135995 34058294

[JEB251082C73] Wouwe, T. V., Ting, L. H. and Groote, F. D. (2022). An approximate stochastic optimal control framework to simulate nonlinear neuro-musculoskeletal models in the presence of noise. *PLoS Comput. Biol.* 18, e1009338. 10.1371/journal.pcbi.1009338 35675227 PMC9176817

[JEB251082C74] Wu, A. R., Simpson, C. S., van Asseldonk, E. H. F., van der Kooij, H. and Ijspeert, A. J. (2019). Mechanics of very slow human walking. *Sci. Rep.* 9, 18079. 10.1038/s41598-019-54271-2 31792226 PMC6889403

[JEB251082C75] Wuehr, M., Schniepp, R., Pradhan, C., Ilmberger, J., Strupp, M., Brandt, T. and Jahn, K. (2013). Differential effects of absent visual feedback control on gait variability during different locomotion speeds. *Exp. Brain. Res.* 224, 287-294. 10.1007/s00221-012-3310-6 23109084

[JEB251082C76] Wuehr, M., Schniepp, R., Schlick, C., Huth, S., Pradhan, C., Dieterich, M., Brandt, T. and Jahn, K. (2014). Sensory loss and walking speed related factors for gait alterations in patients with peripheral neuropathy. *Gait Posture* 39, 852-858. 10.1016/j.gaitpost.2013.11.013 24342450

